# Short- and long-term changes in sugarbeet (*Beta vulgaris* L.) gene expression due to postharvest jasmonic acid treatment - Data

**DOI:** 10.1016/j.dib.2017.02.008

**Published:** 2017-02-09

**Authors:** Lucilene Silva de Oliveira, Karen Klotz Fugate, Jocleita Perruzo Ferrareze, Melvin D. Bolton, Edward L. Deckard, Fernando L. Finger

**Affiliations:** a Departamento de Fitotecnia, Universidade Federal de Viçosa, 36571-000 Viçosa, MG, Brazil; b USDA-ARS, Northern Crop Science Laboratory, 1605 Albrecht Blvd. N., Fargo, ND 58102-2765, USA; c Instituto Federal de Santa Catarina, Campus Lages, Rua Heitor Villa Lobos, 222, São Francisco, Lages, SC 88506-400, Brazil; d Department of Plant Sciences, North Dakota State University, P.O. Box 6050, Fargo, ND 58108-6050, USA

**Keywords:** Plant defense, Root, Storage, Sugar beet, Taproot

## Abstract

Jasmonic acid is a natural plant hormone that induces native defense responses in plants. Sugarbeet (*Beta vulgaris* L.) root unigenes that were differentially expressed 2 and 60 days after a postharvest jasmonic acid treatment are presented. Data include changes in unigene expression relative to water-treated controls, unigene annotations against nonredundant (Nr), Swiss-Prot, Clusters of Orthologous Groups (COG), and Kyoto Encyclopedia of Genes and Genomes (KEGG) protein databases, and unigene annotations with Gene Ontology (GO) terms. Putative defense unigenes are compiled and annotated against the sugarbeet genome. Differential gene expression data were generated by RNA sequencing. Interpretation of the data is available in the research article, “Jasmonic acid causes short- and long-term alterations to the transcriptome and the expression of defense genes in sugarbeet roots” (K.K. Fugate, L.S. Oliveira, J.P. Ferrareze, M.D. Bolton, E.L. Deckard, F.L. Finger, 2017) [1]. Public dissemination of this dataset will allow further analyses of the data.

**Specifications Table**

**Table:** 

Subject area	Biology
More specific subject area	Plant biology
Type of data	Tables
How data was acquired	RNA sequencing using an Illumina, Inc. HiSeq 2000 system
Data format	analyzed
Experimental factors	roots treated with water or 10 µM jasmonic acid
Experimental features	Roots from 16–18 week old plants were submerged for 1 h in water or 10 µM jasmonic acid, and stored at 20 °C and 90% relative humidity for 2 or 60 d.
Data source location	USDA-ARS, Fargo, ND USA
Data accessibility	Data is available with this article

**Value of the data**

• Data will be useful for investigating the effect of jasmonic acid (JA) on gene expression in plants by comparing to similar data sets generated for other plant species and identifying commonly expressed genes.
• Data can be used to infer physiological and metabolic effects of JA which can stimulate new areas of investigation.
• Data can be used to investigate differences in the effect of JA on gene expression between plant species or between plant organs.
• JA-induced defense genes could be further investigated for their ability to protect sugarbeet root against insects or pathogens.
• Data can be used to investigate short-term and long-term JA treatment effects by comparison of data collected at 2 and 60 days post-treatment.

## 1 Data

Data on sugarbeet root unigenes that were differentially expressed due to a postharvest jasmonic acid (JA) treatment are presented. Data identify unigenes that were differentially expressed 2 and 60 d after JA treatment. Data include the logarithm of the fold change in expression due to JA treatment, relevant statistics related to changes in expression, unigene annotations generated by BLASTx search against nonredundant (Nr), Swiss-Prot, Clusters of Orthologous Groups (COG), and Kyoto Encyclopedia of Genes and Genomes (KEGG) protein databases, and Gene Ontology (GO) annotations (Tables 1 and 2). In addition, differentially expressed genes with putative defense functions were identified, compiled, and further annotated by comparison to the sugarbeet genome [2] (Tables 3 and 4).

## 2 Experimental design, materials and methods

### 2.1 Plant material and postharvest treatment

Sugarbeet taproots were produced and harvested as previously described [1]. Freshly harvested taproots were submerged in water or aqueous 10 μM JA (Cayman Chemical, Ann Arbor, MI, USA) for 1 h at room temperature and stored at 20 °C and 90% relative humidity for up to 60 d in a controlled environment chamber (Conviron, model MTR30, Winnipeg, Canada). Root samples were collected 2 and 60 d post-treatment by collecting tissue from the main portion of the taproot, free of crown or tail tissue, with the epidermis and approximately 2 mm of subepidermal tissue excluded. Samples were flash frozen in liquid N_2_, lyophilized, ground to a powder, and stored at −80 °C. Individual taproots were the experimental unit with four replicates per treatment per time point. Experiment was repeated twice.

### 2.2 RNA isolation and sequencing

Replicates for each time point and treatment within an experiment were pooled using an equal weight of lyophilized tissue from each taproot. Total RNA was extracted from these pooled samples (20 mg) using an RNeasy Plant Mini Kit (Qiagen, Valencia, CA, USA) with an on-column DNase digestion. RNA quality was confirmed using an Agilent Technologies 2100 Bioanalyzer (Pal Alto, CA, USA). RNAs were converted to cDNAs and sequenced. cDNA library preparation and sequencing were performed by BGI Americas (Cambridge, MA, USA). cDNAs were sequenced using an Illumina, Inc. HiSeq 2000 system (San Diego, CA, USA). Repetitions of the experiment served as replicates.

### 2.3 Bioinformatics

Raw sequence data was cleaned to remove reads with adapters, reads with >10% unknown bases, and low quality reads. Clean reads were mapped to a sugarbeet reference transcriptome [3] using SOAPaligner/soap2 [4]. Mismatches of no more than 2 bases were permitted. Differential gene expression between water and JA-treated roots at 2 d post-treatment and between water and JA-treated roots at 60 d post-treatment were determined using RobiNA software [5]. Only unigenes with an absolute value of log_2_ (fold change) ≥1 and a false discovery rate (FDR) ≤0.001 were considered differentially expressed. Annotations to the sugarbeet genome [2] were obtained by BLASTn search of unigene sequences [6].
