# Supplementary material for: Short- and long-term changes in sugarbeet (Beta vulgaris L.) gene expression due to postharvest jasmonic acid treatment - Data
Source: Data Brief. 2017 Feb 9;11:165–8. doi: 10.1016/j.dib.2017.02.008 (PMC5312494; doi:10.1016/j.dib.2017.02.008)
Supplement: Supplementary file 1 — Supplementary material [file mmc1.docx]

**Manuscript Title:** Short- and long-term changes in sugarbeet (Beta vulgaris L.) gene expression due to postharvest jasmonic acid treatment - Data

**Authors:** Lucilene Silva de Oliveira, Karen Klotz Fugate, Jocleita Perruzo Ferrareze, Melvin D. Bolton, Edward L. Deckard, and Fernando L. Finger

The authors certify that they have NO affiliations with or involvement in any organization or entity with any financial interest (such as honoraria; educational grants; participation in speakers’ bureaus; membership, employment, consultancies, stock ownership, or other equity interest; and expert testimony or patent-licensing arrangements), or non-financial interest (such as personal or professional relationships, affiliations, knowledge or beliefs) in the subject matter or materials discussed in this manuscript.
